# Asymmetrical barcode adapter-assisted recovery of duplicate reads and error correction strategy to detect rare mutations in circulating tumor DNA

**DOI:** 10.1038/srep46678

**Published:** 2017-05-02

**Authors:** Jinwoo Ahn, Byungjin Hwang, Ha Young Kim, Hoon Jang, Hwang-Phill Kim, Sae-Won Han, Tae-You Kim, Ji Hyun Lee, Duhee Bang

**Affiliations:** 1 Department of Chemistry, Yonsei University, Seoul, Korea; 2 Cancer Research Institute, Seoul National University, Seoul, Korea; 3 Department of Molecular Medicine and Biopharmaceutical Sciences, Graduate School of Convergence Science and Technology, Seoul National University, Seoul, Korea; 4 Department of Internal Medicine, Seoul National University Hospital, Seoul, Korea; 5 Department of Clinical Pharmacology and Therapeutics, College of Medicine, Kyung Hee University, Seoul, Korea

## Abstract

Deep sequencing is required for the highly sensitive detection of rare variants in circulating tumor DNA (ctDNA). However, there remains a challenge for improved sensitivity and specificity. Maximum-depth sequencing is crucial to detect minority mutations that contribute to cancer progression. The associated costs become prohibitive as the numbers of targets and samples increase. We describe the targeted sequencing of *KRAS* in plasma samples using an efficient barcoding approach to recover discarded reads marked as duplicates. Combined with an error-removal strategy, we anticipate that our method could improve the accuracy of genotype calling, especially to detect rare mutations in the monitoring of ctDNA.

The advent of massively parallel DNA sequencing has revolutionized the field of genomics and transformed human medical research. The cost of sequencing has decreased rapidly^1^ since 2005, which has enabled researchers to explore many basic questions in natural science. Currently, the non-invasive detection of circulating tumor DNA (ctDNA) has great potential to be the ‘holy grail’ of early cancer detection. Cost could be a substantial concern in large-scale projects, however, because great sequencing depth must be achieved to reliably detect rare alleles in ctDNA samples.

It is a major challenge to distinguish true variants from background noise for rare variant discovery^2^,^3^, because the error rate ranges from 1 to 10%, which prohibits deconvolution of the clonal structure of tumor genomes. In addition, the heterogeneity of mutations within populations of rare cells in liquid biopsy samples, such as plasma^4^ or saliva^5^, make it difficult to distinguish between sequencing-related errors and true somatic mutations originating from tumors. To overcome this issue, high-depth sequencing is frequently used to detect rare variants^6^,^7^,^8^. As a quality-control measure for next-generation sequencing (NGS) data, duplicate reads are usually discarded to allow computational ease of downstream analyses and to mitigate PCR amplification biases. However, removal of duplicate reads could result in genotyping errors in libraries constructed from only a small amount of input material because of the inherently imbalanced nature of the sequencing coverage^9^.

One potential solution for tracking duplicate sequences is molecular barcoding, which could enhance the accuracy of detection by distinguishing real variants from artifacts generated during PCR. Various barcoding approaches have been implemented to quantify individual molecules^10^,^11^ and have allowed actual somatic mutations to be distinguished from background artifacts. Although the ‘duplex’ barcoding strategy yields significantly lower error rates^10^,^12^, the method is suboptimal because the required sequencing depth is high. To overcome this limitation, a hybrid approach that combines a statistical error-suppression algorithm with barcodes that track both single-stranded and double-stranded DNA was recently introduced^13^.

Here, we introduce an asymmetrical sequencing adapter design with the hybridization capture of a small genomic region (the *KRAS* gene) to recover duplicate reads for the quantification of individual molecules. Our experimental design enabled us to demultiplex samples using the standard Illumina P5 index sequence as well as to barcode double-stranded molecules using an 8-bp random sequence inserted in the Illumina P7 index position. We aligned the demultiplexed samples to the reference sequence, marked duplicate reads as candidates for recovery, and recovered and re-aligned reads that were initially discarded as duplicates. Finally, we smoothed the background errors using Bayesian likelihood estimation. Our simple yet efficient strategy enabled the accurate detection of rare mutations in case-control studies as well as in the non-invasive screening of ctDNA.

## Results

### Barcoding using an asymmetrical sequencing adapter

**Figure 1:**
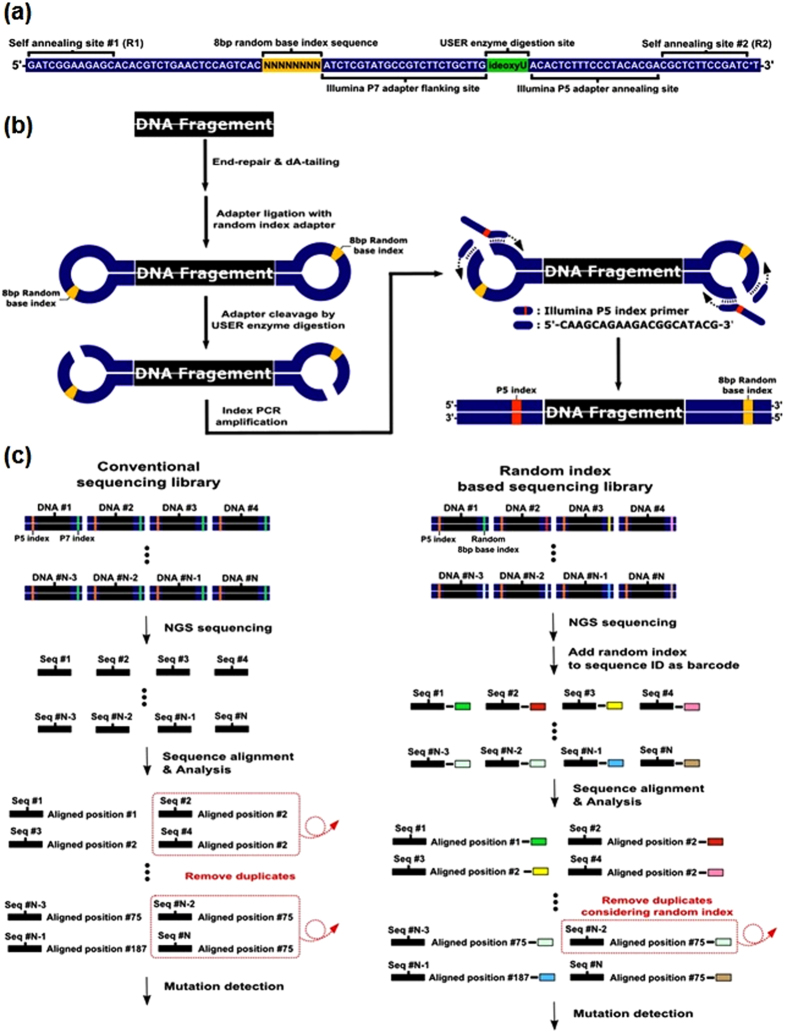
A schematic of the asymmetrical barcoding method. (**a**) Design of the asymmetrical random index sequencing adapter oligonucleotide. Self-annealing sites 1 and 2 are referred to as R1 and R2, respectively. (**b**) Sequencing library-preparation scheme using the asymmetrical random index sequencing adapter. The self-annealed barcodes in **a** were used in the adapter ligation step and subsequently amplified using the P7 flanking primer and the P5 index primer. (**c**) Comparison of the removal of duplicate candidates with a conventional duplication removal strategy. Molecules with the same barcodes and the same start/end positions were considered true duplicate pairs.

We generally defined duplicate reads as read pairs with identical external mapping coordinates. To recover discarded duplicate reads, we designed an experiment using an asymmetrical barcode sequence to distinguish true reads from false duplicate PCR reads^14^,^15^ (Fig. 1a). We assigned an 8-bp random ‘N’ barcode sequence to each fragmented DNA molecule by adjusting the standard Illumina sequencing adapter at the P7 index position. Prior to the preparation of the sequencing library, we designed adapter oligonucleotides that self-annealed to produce loop-form adapters. We then attached the self-annealed sequencing adapters to both ends of the randomly sheared genomic DNA molecules using an adapter ligation step (Fig. 1b). After USER enzyme digestion, the ligated adapters created an asymmetric Y-adapter form, which we used to perform PCR with the flanking P7 and P5 primers (to provide a sample barcode for the sorting of data from different samples). Details of the sequencing library preparation and the experimental conditions are provided in the Materials and Methods section.

### Detection of rare mutations in patients with colorectal cancer

To test the abilities of the asymmetric barcode adapter to recover sequencing depth and detect rare mutations in a clinical application, we constructed a sequencing library using plasma samples from five patients with colorectal cancer and focused on a *KRAS* mutation known to be prevalent in such cases^16^,^17^,^18^. We validated the *KRAS* mutation in the five patients by Sanger sequencing using both tumor and whole blood samples. The detailed protocol and primer sequences used for the Sanger sequencing are provided in the Materials and Methods section.

We generated an NGS library based on the three samples by applying our asymmetrical sequencing adapter design with an Illumina HiSeq 4000 system. After discarding low-quality reads, we aligned the reads to the reference genome (GRCh37/hg19) and marked duplicate reads as candidates for recovery. Next, we re-evaluated the discarded reads using the 8-bp barcode composition and aligned the positions to identify the true duplicates (Fig. 1c, Supplemental Fig. 1). We found that these UMI counts were overestimated as they far exceed the upper bound of given input amount. We therefore calculated hamming distances between UMIs within a hamming distance of two or less and retained only the UMI with the highest read count within clusters. In all samples, 25% of original barcodes were discarded and the background error rate dropped 30% on average.

**Figure 2:**
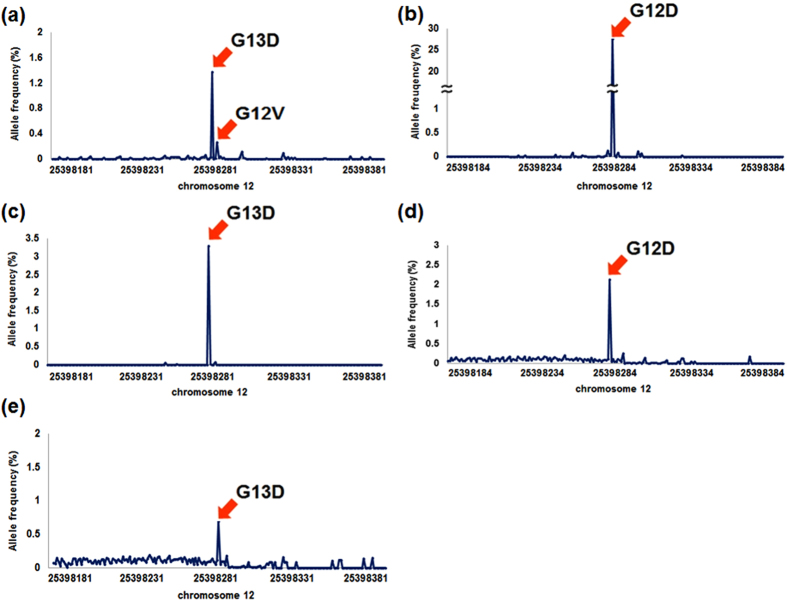
Analysis of the performance of the barcoding strategy in five clinical samples. The observed allele frequency distributions after error correction for each sample are plotted. The central peaks (amino acid numbers 12 and 13) of chromosome 12 in the graph are the primary mutations detected in the sample. The major-mutation peaks of samples (**a**) ctDNA1, (**b**) ctDNA2, (**c**) ctDNA3, (**d**) ctDNA4 and (**e**) ctDNA5 are indicated by the arrows. The allele frequency was calculated within a specific 200-bp region of chromosome 12.

As a result, we observed a significant increase in the mean sequencing depth of each sample (average 2-fold depth increase in the target region), which could enhance the reliability of rare variant detection in clinical samples (Supplemental Fig. 2). Next, to remove background calls, we used the asymmetric barcode adapter and an error correction strategy. Most of the background errors entailed variant-allele frequencies below 0.2%. A large fraction of these false calls were removed by statistical error correction (Fig. 2, Material and Methods). We found that the driver-mutation peaks displayed similar frequencies (average standard deviation = 0.12) after applying our statistical filtering process. Notably, in the ctDNA1 sample harboring the main driver-mutant peak of the G13D mutation, we also detected the G12V mutation in *KRAS* with a variant-allele frequency of approximately 0.3% (Fig. 2a). Further examination of the reads bearing the G12V mutation confirmed that the reads did not originate from duplicates (Supplemental Fig. 3). We performed Sanger sequencing of the *KRAS* gene from tumor and normal tissues, and confirmed that the G12V mutation was present only in the tumor tissue (Supplemental Fig. 4). We analyzed the other ctDNA samples in the same manner and detected clear *KRAS* mutation peaks (Fig. 2b–e). Then, we assessed the sensitivity and specificity of our method using all five plasma samples. We applied the ctDNA index^19^ as described previously for receiver operating characteristic analysis and achieved an area under the curve of 0.99 after applying the error correction (Supplemental Fig. 5 and Materials and Methods).

We then analyzed the recovered fraction of reads marked as duplicates for varying amounts of ctDNA input (Supplemental Table 1). We discovered that as the initial number of haploid genome equivalents increased, the number of duplicate reads decreased, despite a similar number of sequencing reads for all of the samples. We reasoned that the proportion of duplicate reads would decrease as the number of distinct molecules increased. To further explore this and other factors affecting the duplicate fraction, we conducted a simulation study to validate our experimental results.

### Simulation of the read duplication fraction

**Figure 3:**
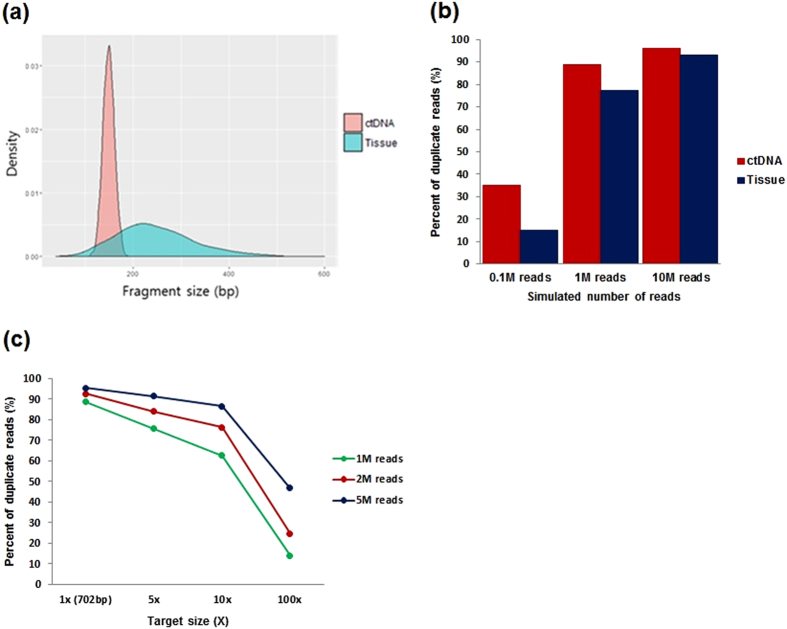
Simulation of sequencing reads in the small target region (*KRAS* gene, five exonic regions) and the impact of the barcoding strategy. (**a**) Size distribution of the sequencing data from the ctDNA and tissue DNA. Two different distributions were used to model each sample type. (**b**) Simulation of the estimated duplication fraction according to the sample type and number of sequencing reads. The duplication rate of the ctDNA was higher than that of the tissue DNA. (**c**) Simulation of the duplication rate according to the target size (assuming 0.1% sequencing errors).

To examine the fraction of sequencing reads that were regarded as PCR duplicates in the ctDNA sequencing data, we designed a computational simulation to predict the fraction of duplicate reads that would result during sequencing given variables such as the number of sequencing reads and number of distinct molecules. To compare relative numbers of duplicate reads (Fig. 3a), we simulated NGS reads for ctDNA and sheared tumor DNA using a given number of sequencing reads based on empirical distributions of read lengths. We used a Poisson distribution for the ctDNA because the read length distribution revealed a sharp peak around ~180 bp, with little variance. Conversely, we used a negative binomial (NB) model to fit the tissue DNA length distribution because of the increased flexibility in parameter controls.

We simulated read duplication considering two possible sources of read duplication. The first source of duplication, which is the most common, is amplification bias. The second source of duplication is sampling or alignment duplication triggered by random fragmentation (see Materials and Methods for details). We conducted our simulation assuming a 0.1% substitution-error rate (major errors in Illumina sequencing) in the sequencing reads. We observed an increase in the duplication fraction as the number of simulated reads increased; however, the impact of the two sources of duplication was different between the tissue and ctDNA samples. Consistent with the current notion, PCR amplification was the greatest source of duplication in both samples. However, sampling-induced duplication contributed to the duplication rate was non-negligible in the ctDNA. Notably, the effect of sampling-induced duplication was more pronounced in the ctDNA sample, which is most similar to the actual samples used in the present study (Fig. 3b, see also Materials and Methods).

From our simulations, we concluded that as the insert became shorter and less variable, the number of unique reads that could cover the target site declined. In addition, as the number of unique molecules increased, the number of duplicate reads that could be observed declined. To investigate the possibility of extending our experiment to multi-target regions, we examined the effect of increasing the size of the target region. As expected, the duplication rate decreased as the target region increased, implying a reduced frequency of sampling-induced duplication (Fig. 3c).

### Targeted sequencing of rare variants from admixture samples

To assess the analytical sensitivity of the asymmetric barcode adapter, we constructed an additional NGS library using genomic DNA from the CEU HapMap sample NA12878 as a reference and a colorectal cancer cell line (SW480) known to harbor the *KRAS* G12V mutation as a positive control. We generated a sequencing library from these samples by applying the asymmetrical sequencing adapter design and performed Illumina paired-end sequencing (HiSeq 4000). Next, we created a shuffled library of admixtures containing 1%, 0.5%, and 0.25% of the mutation-bearing SW480 DNA data (Supplemental Fig. 6). After the initial processing of the primary sequences as well as quality trimming and misalignment error removal, we calculated the sequencing depths in the target region. We found that the mean depth after barcode-assisted read recovery increased an average of 2.7-fold (Supplemental Fig. 7), which could facilitate the detection of rare mutations. Further removal of background mutations using statistical error correction enabled reliable detection of the G12V mutation at the expected frequency in each sample, even when the frequency was as low as 0.25% (Supplemental Fig. 8).

## Discussion

False-positive mutations based on sampling bias and sequencing errors pose a challenge to the identification of mutations associated with therapeutic targets. In addition, false positives due to factors such as *ex vivo* oxidative damage can arise during library preparation^20^,^21^. Our data revealed a particularly high frequency of G:C > A:T transition errors, which were in line with previous reports^13^,^22^. The majority of the alleles found to be reduced after error correction were transition-substitution alleles (Supplemental Fig. 9). These factors might complicate estimations of allele frequency and copy number variation in gene-expression experiments. Accordingly, high-depth sequencing is commonly used to reduce the noise due to background errors and enable the reliable detection of clinically relevant mutations.

Duplicates are often removed in the analysis of NGS data to correct the read-count bias induced by preferential amplification. However, loss of sequencing depth inevitably occurs, because sequenced DNA fragments with the same alignment position and length are indistinguishable from one another. Unlike the detection of germline variants in monoclonal samples, the evaluation of rare clonal mutations in ctDNA studies requires high-depth sequencing. Thus, sequencing costs increase in an effort to achieve the desired sequencing depth^23^. In addition, unexpected depth loss might impact the sensitivity of the detection limit. Hence, the common practice of removing duplicates can only be justified when sampling coincidence is unlikely and the sequencing depth is low. Nevertheless, no prior studies have attempted a systematic evaluation of the issue. It is critical to estimate the extent of the duplication rate in such scenarios, because the ultimate goal of the sequencing is to obtain a depth of coverage that is adequate for the detection of mutations in various types of samples with reasonable associated costs.

In our study, simulations revealed that a key aspect of the analysis of duplication rates is variation in the insert size. We found that for data with minimal associated variance, such as the ctDNA library, duplicate reads should be removed with great caution, because low-input DNA with high-coverage sequencing leads to more PCR amplification-induced duplicates. The findings from our simulation experiments might vary somewhat from those of actual data, because the actual sequencing error rate is variable and the fragmentation pattern more stochastic. However, it is clear that the impact of the duplicate-removal process samples like ours would not be negligible.

To overcome this problem, we designed a novel barcode adapter to recover reads commonly marked as duplicates. Generally, a sequencing library adapter is used with different types of sequencers for demultiplexing, but not for distinguishing DNA fragments. We modified one side of the sequencing adapter by incorporating random ‘N’ sequences. The diversity of the 4^8^ (random 8-bp) nucleotide sequences provided barcodes that were sufficiently distinguishable in clinically relevant amounts of DNA (~20 ng). Our strategy allowed us to simultaneously demultiplex the samples and quantify the double-stranded molecules. Unlike polymerase extension using degenerate primers and a synthetic template^11^,^24^, which limits the diversity of the tags produced, our method generated each molecular tag as a single unit using a balanced mix of oligonucleotides, which provided the desired diversity. This method also allowed us to maximize the informative read length, and thus the sequencing throughput, because preparation of the asymmetric adapter-based sequencing library does not involve an additional adapter annealing sequence. Our strategy was developed based on Illumina’s hairpin adapter by adding a degenerate 8-bp N sequence at the P7 index position, which caused no drop in the library quality. The retention of the original Illumina adapter annealing-arm sequences for the Y-adapter rendered greater ligation efficiency. The use of our barcode system could be extended to the detection of rare clonal variants in more challenging formalin-fixed, paraffin-embedded samples.

One of the other potential applications for the barcode adapter is detection of changes in copy number. Similar to the detection of rare somatic nucleotide variants, the overcorrection of read counts would be detrimental in copy number detection, which is based on the enumeration of the sequencing depth of the target regions^25^. Our simulation study demonstrated that the impact of duplication removal was substantial following the high-depth sequencing of libraries with smaller insert sizes and variances, such as the fragmented ctDNA library. A quantitative understanding of the duplicate rates in simulations of ctDNA and tissue samples leads us to conclude that deduplication is justified only when the sequencing depth is moderate and the insert size variance is high, and its use should be carefully considered when performing high-depth sequencing experiments.

In summary, our barcoding approach improved the sensitivity of rare variant detection, which enabled the detection of rare alleles in ctDNA samples at a reasonable cost (Supplemental Table 2). We anticipate that our simple barcoding approach, in conjunction with a statistical error correction strategy, could be generally applicable to various NGS-based studies for the accurate quantification of low-frequency alleles and copy number variants.

## Materials and Methods

### Random index-adapter synthesis and adapter folding for sequencing library preparation

Random index adapters consisted of an 8-bp random barcode at the P7 index position. The oligonucleotides (IDT, USA) for the random index adapter were designed as previously discussed (Fig. 1a). Synthesized adapter oligonucleotides were diluted to a concentration of 100 μM and annealed to form a stem-loop adapter following incubation at 95 °C for 3 min and ramping of 0.1 °C per second to 37 °C. The adapter ligation mixture contained 5 μl 100 μM stem-loop folded adapter oligonucleotides, 5 μl T4 DNA ligase buffer (NEB, USA), and 40 μl nuclease-free water. Folded adaptors were used in the adaptor ligation step during the sequencing library preparation.

### Preparation of clinical samples from patients with colorectal cancer

Blood samples were collected from patients with metastatic colorectal cancer receiving chemotherapy at Seoul National University Hospital. From the patients who agreed to voluntarily donate their blood samples for research purposes, 4~6 ml whole blood was collected into EDTA tubes during routine phlebotomy. Plasma was separated by centrifugation with Ficoll solution at 2,000 rpm for 15 min and transferred into micro-centrifuge tubes. Then, the plasma was centrifuged at 13,000 rpm for 10 min to remove cell debris. The supernatant was stored at −80 °C before extraction. The entire separation protocol was performed within 20 min of collection to prevent the degradation of cell-free DNA. Additional clinical information including gender, smoking history, and age was collected (Supplemental Table 3). All patients provided written informed consent before undergoing any study-specific procedures including phlebotomy. The study was approved by the Institutional Review Board of Seoul National University Hospital [IRB number: 1407–060–592] and was conducted in accordance with the Helsinki Declaration.

### Sanger validation of the *KRAS* mutation

The primer pair used for the validation sequencing of the *KRAS* mutation was designed with the Primer3 program^26^. The sequences of the primer pair were as follows: ‘f-CTGTATCAAAGAATGGTCCTGC’ and ‘r-CACTATCAAATACTCCACCAGTACC.’ Genomic DNA (gDNA) was extracted from plasma and from normal and tumor tissues obtained from the patients using the DNeasy Blood & Tissue DNA purification kit (Qiagen, USA). Target PCR amplification was carried out using a mixture of 10 μl Taq polymerase 2X Master Mix (iNtRON Biotechnology, Inc., Korea), 8 μl nuclease-free water, 10 ng gDNA, and 1 μl each primer. The PCR conditions included an initial denaturing step of 3 min at 95 °C followed by 40 cycles of 95 °C for 30 s, 58 °C for 30 s, and 72 °C for 30 s and a final extension at 72 °C for 10 min. The confirmed somatic mutations were then compared with the variants noted in the ctDNA data.

### Sequencing library preparation and *KRAS* gene-target capture

Genomic DNA was extracted from the cell lines using the DNeasy Blood & Tissue Kit (Qiagen, USA). The QIAamp Circulating Nucleic Acid Kit (Qiagen, USA) was used to extract ctDNA from 2–3 ml plasma collected from the patients with colorectal cancer. To make the DNA fragment size similar to that of the plasma DNA (for the dilution experiment), shearing (Covaris, USA) was performed for 55 s in a reaction volume of 130 μl. DNA from the plasma samples was used directly for sequencing library preparation. The extracted DNA was end repaired, dA-tailed using the *SPARK*™ DNA Prep kit (Enzymatics, USA), and ligated using the folded asymmetrical barcode adapter, which was accompanied by USER enzyme (NEB, USA) digestion in accord with standard Illumina sequencing library-preparation protocols. After digestion with the USER enzyme, the samples were PCR amplified using the P5 index primer and the P7 adapter flanking-sequence primer (‘CAAGCAGAAGACGGCATACG’). The PCR mixture consisted of 25 μl KAPA 2X polymerase (Kapa Biosystems, USA), 16 μl nuclease-free water, 5 μl USER-digested product, and 2 μl each primer. The PCR conditions included an initial denaturing step of 3 min at 95 °C followed by eight cycles of 95 °C for 30 s, 52 °C for 30 s, and 72 °C for 30 s and a final extension at 72 °C for 10 min. Each reaction was then purified using AMPure XP beads (Beckman Coulter, USA). *KRAS* target capture was performed according to the manufacturer’s protocol using a *KRAS* capture kit (Celemics, Inc., Korea), which targets five exonic regions of *KRAS* (Supplemental Table 4). The adapter-attached library was denatured at 95 °C for 5 min and then incubated at 65 °C before the addition of adaptor-specific blocker DNA in hybridization buffer accompanied by the customized-baitset reagent, Cot, and salmon sperm. The library sample was hybridized for 24 h, and off-target library was removed using T1 Magnetic Beads. Then, the post PCR amplification step was performed using the target-captured library. The PCR mixture consisted of 25 μl KAPA 2X polymerase (Kapa Biosystems, USA), 20 μl target-captured sample, and 2.5 μl each primer (‘f-AATGATACGGCGACCACCGAG,’ ‘f-CAAGCAGAAGACGGCATACG’). The PCR conditions included an initial denaturing step of 3 min at 95 °C followed by 16 cycles of 95 °C for 30 s, 52 °C for 30 s, and 72 °C for 30 s and a final extension at 72 °C for 10 min. After purification using AMPure XP beads, the samples were sequenced using a 150 bp paired-end Illumina^®^ HiSeq 4000 platform (Theragen Etex Co., Ltd., Korea).

### Sequencing and data processing

The sequencing reads with an average Phred base quality score (across entire read) less than 30 were filtered out. The filtered reads were then aligned to the reference genome using NovoAlign (v. 2.07.18, Novocraft Technologies, Malaysia). The Picard Mark Duplicates tool (v.1.128, Broad Institute, USA) was used to mark the candidate duplicate PCR read pairs. We called those reads set I and the unmarked reads set II. We rescued reads from set I reads when the alignment position and random index sequence were exactly the same but the 8-bp barcode sequence was different. The set of rescued reads was joined with the reads from set II and included in the subsequent re-alignment and mutation-detection steps.

### Error correction analysis

At the mutation-detection step, the high-confidence, paired, aligned reads (minimum Phred base quality score of 35 in reads) were combined (with at least a 50-bp overlap) into a single consensus reads set to reduce sequencing errors. Non-consensus positions between the forward and reverse reads were labeled as ‘N’.

Mutant alleles were detected using a Bayesian methodology based on prior cancer-specific expectations (based on TCGA MAF files downloaded from the National Institutes of Health website). The probability of observing genotype G in data D from an individual locus was expressed as:


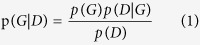


where P(G) was the prior expectation, and p(D|G) was the likelihood of observing the genotype at a locus in a diploid genome (assuming two alleles), the calculation of which can be derived from the error probability matrix. The error probability matrix p(D|G) for all 10 genotype combinations was calculated as the observed sequenced nucleotide versus the actual reference nucleotide in the control samples. The control samples refer to all five plasma DNA samples used to estimate the background distribution. 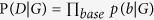, the probability of each base in a given genotype, was defined as 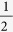
[(*p(b*|*A*1) + *p(b*|*A*1)], where G = {A1, A2} represented two alleles. Finally, the posterior probability p(G|D) was emitted to disk, and an alternate base in a given genotype was set as background error if the specific genotype combination did not exceed a certain threshold value (0.1 for this study, a user-tunable value). After removing erroneous bases using this soft cut-off strategy, we used somatic lesions as candidate reporters (confirmed by the Sanger sequencing of matched germline samples) to calculate the ctDNA index as in Newman *et al*.^19^. Briefly, multiple reporters for each individual probability were combined into single *P*-value. First, the allele fraction is adjusted incorporating position-specific error rate and the selector-wide (target capture panel) background distribution. Then, using 1,000 iterations of Monte Carlo simulation, these adjusted mean SNV fraction were compared against the null distribution across the selector (target capture panel). All 5 distinct SNVs found in 5-target region (2.2 median SNVs per patient) were validated by Sanger sequencing and independent target capture sequencing in matched tumor samples. ROC analysis was performed as in Supplementary Fig. 6 in original CAPP-Seq paper^19^ which combines all patient reporters.

### Simulation of the read duplication fraction of sequencing data

To calculate the relative read duplication fraction that corresponded to a specific number of sequencing reads, all of the counts were modeled based on an NB distribution (tissue DNA) or a Poisson distribution (ctDNA). We adjusted the mean (Poisson) and dispersion parameters (NB) for the two distributions, which were estimated from actual data. The distribution of the virtual sequencing fragment size is shown in Fig. 3a.

Specifically, we modeled the duplicate rate as the product of the amplicon duplication rate and the alignment duplication rate as follows. Let ‘N’ be the number of unique molecules and ‘m’ be the number of sequencing reads. If ‘C’ is the coverage or the number of amplicons in the library, then C_i_ is assumed to be drawn from a Poisson distribution (mean:λ). Thus, for a sufficiently large N, the total number of libraries (L) is N × λ.

For number of sequencing reads ‘m,’ each molecule ‘i’ can be sampled from a Bernoulli distribution (X_i_ = 1 if C_i_ has been sampled at least once, otherwise X_i_ = 0). Then,


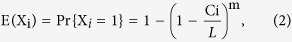


which can be approximated as


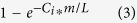



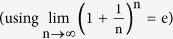
). Let 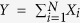 be the total C_i_ sampled from the library, then the amplicon duplication (D_A_) rate is


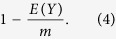


For alignment duplication, we defined ‘m’ as the number of read pairs and X_ij_ as an indicator function [X_ij_ = 1 if at least one reads pair was mapped to the position (i, j), otherwise Xij = 0]. Let R be the reference length and I_k_ be the distribution of insert sizes. Calculations similar to those outlined above lead to:


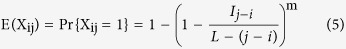


As mentioned above, I_k_ (k = j − i) is a density function of either a Poisson or NB distribution. Therefore, the sampling coincidence (D_s_) is given as:


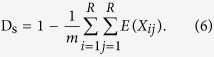


## Additional Information

**How to cite this article:** Ahn, J. *et al*. Asymmetrical barcode adapter-assisted recovery of duplicate reads and error correction strategy to detect rare mutations in circulating tumor DNA. *Sci. Rep.* **7**, 46678; doi: 10.1038/srep46678 (2017).

**Publisher's note:** Springer Nature remains neutral with regard to jurisdictional claims in published maps and institutional affiliations.

## Supplementary Material

Supplementary Information
