# Supplementary material for: Asymmetrical barcode adapter-assisted recovery of duplicate reads and error correction strategy to detect rare mutations in circulating tumor DNA
Source: Sci Rep. 2017 May 2;7:46678. doi: 10.1038/srep46678 (PMC5411960; doi:10.1038/srep46678)
Supplement: Supplementary Information [file srep46678-s1.pdf]

Supporting Information

Asymmetrical barcode adapter-assisted recovery of duplicate reads and error correction strategy to detect rare mutations in circulating tumor DNA

Jinwoo Ahn[1,*], Byungjin Hwang[1,*], Ha Young Kim[1,*], Hoon Jang[1], Hwang-Phill Kim[2,3], Sae-Won Han[2,4], Tae-You Kim[2,3,4], Ji Hyun Lee[5,#], Duhee Bang[1,#]

[1]Department of Chemistry, Yonsei University, Seoul, Korea

[2]Cancer Research Institute, Seoul National University, Seoul, Korea

[3]Department of Molecular Medicine and Biopharmaceutical Sciences, Graduate School of Convergence Science and Technology, Seoul National University, Seoul, Korea

[4]Department of Internal Medicine, Seoul National University Hospital, Seoul, Korea

[5]Department of Clinical Pharmacology and Therapeutics, College of Medicine, Kyung Hee University, Seoul, Korea

[#]Corresponding authors: duheebang@yonsei.ac.kr, hyunihyuni@khu.ac.kr

Supplemental Figure 1. Overview of the analysis pipeline. **(a)** Conventional sequencing data analysis pipeline. **(b)** Sequencing analysis pipeline with sequencing data prepared using our proposed asymmetric barcode adapter. Duplicate reads from step (A) were recovered considering both random 'N' barcode identity and aligned position.

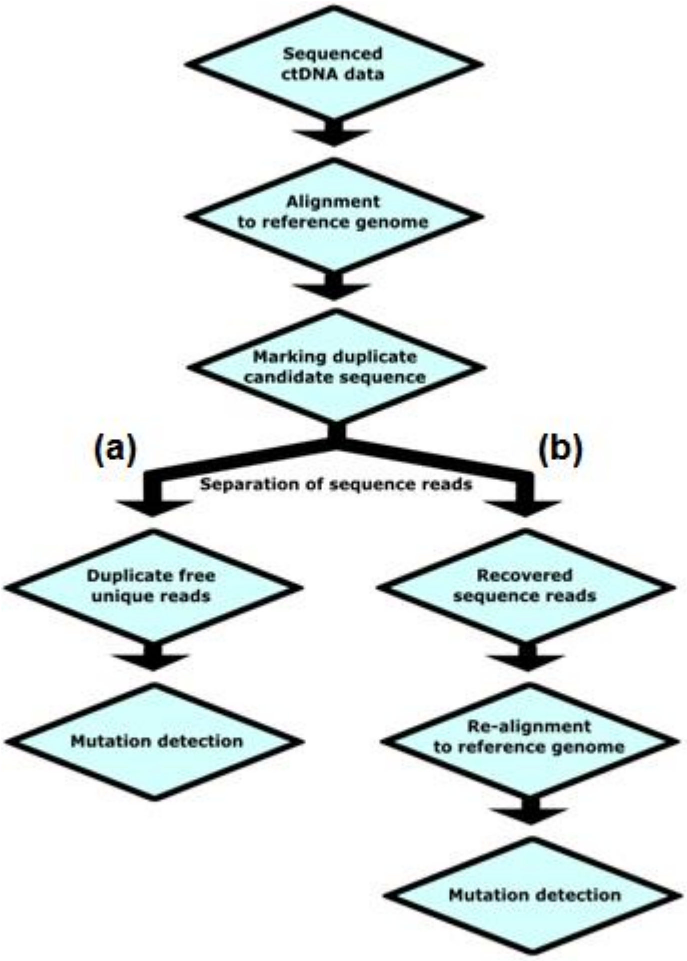

Supplemental Figure 2. Recovery of the depth of coverage in clinical colorectal cancer plasma samples after applying the barcode and statistical error correction.

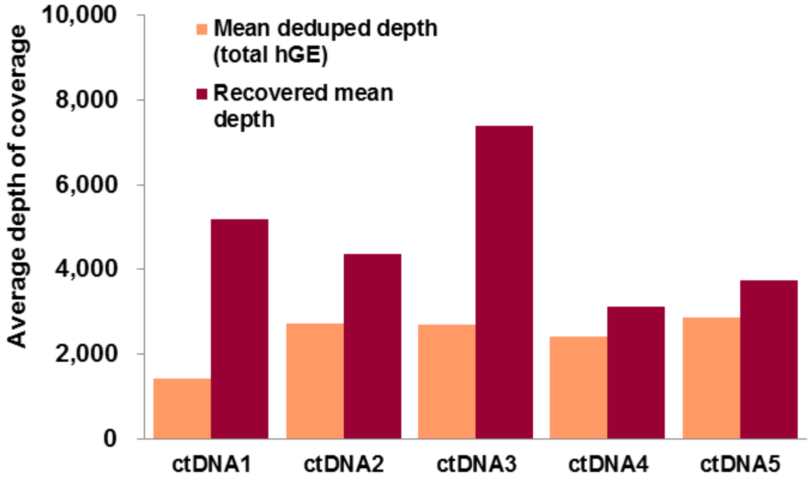

Supplemental Figure 3. The barcode complexity estimation of the reads aligned to the 12th amino acid position (variant allele frequency < 0.3%) of the *KRAS* gene in ctDNA1 sample. The x-axis refers to the reads (barcodes) containing the mutant allele (G12V) and the y-axis refers to the identical fraction between pairwise barcodes [i.e., if 2 out of 19 barcodes share 4-bp (50%, same_4) sequences, the identical fraction for Bar#1 is 10.5%].

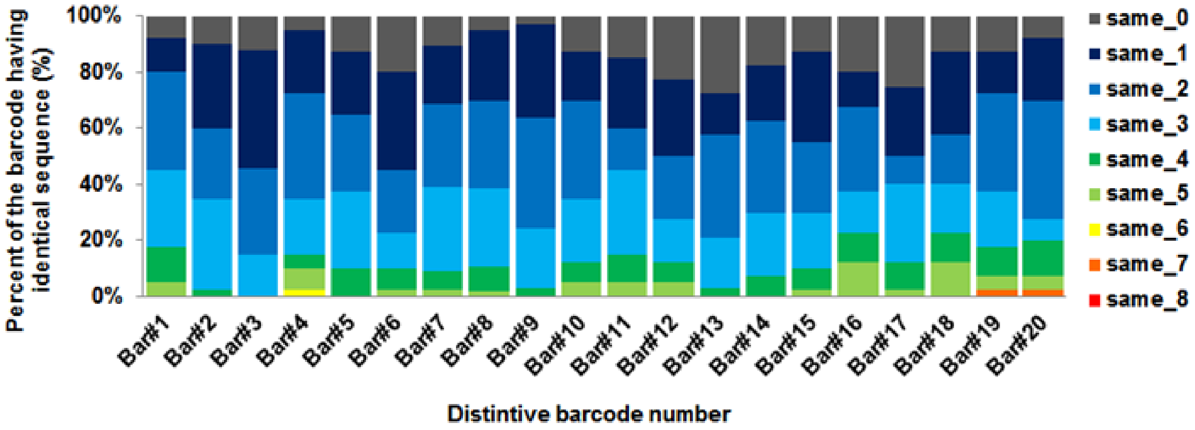

Supplemental Figure 4. Sanger validation result from tumor and normal tissue from ctDNA1 patient with *KRAS* mutations. To note, low frequency variant (G12V), validated by Sanger sequencing, was also called as mutation in NGS data after removing background errors.

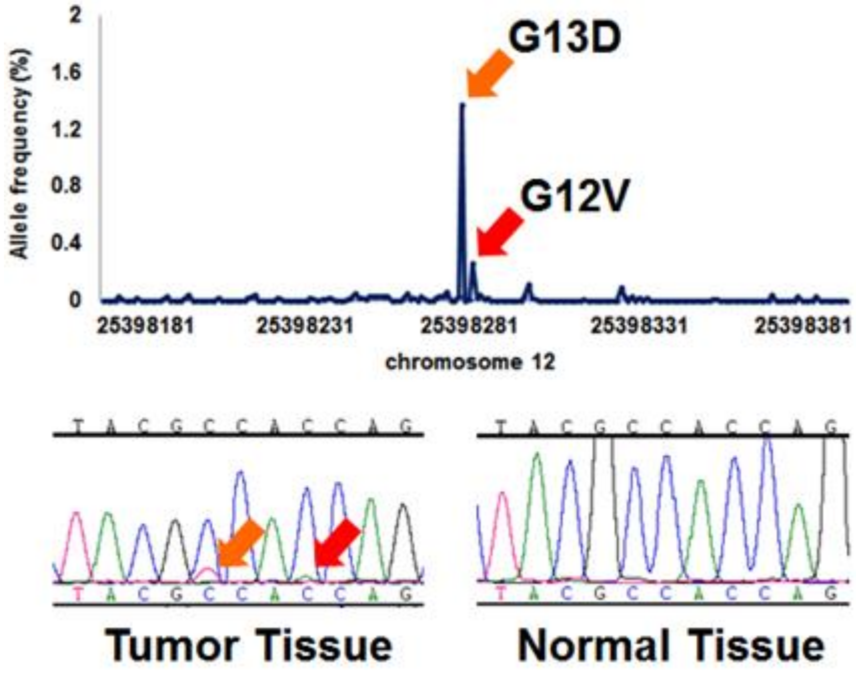

Supplemental Figure 5. Sensitivity and specificity analysis. Sensitivity and specificity analysis using Receiver Operating Characteristic (ROC) before (control) and after error-correction strategy applied. Area Under the Curve (AUC) values were 0.95 and 0.99 respectively.Statistical significance testing was conducted using Wilcoxon's test (*P* < 0.001).

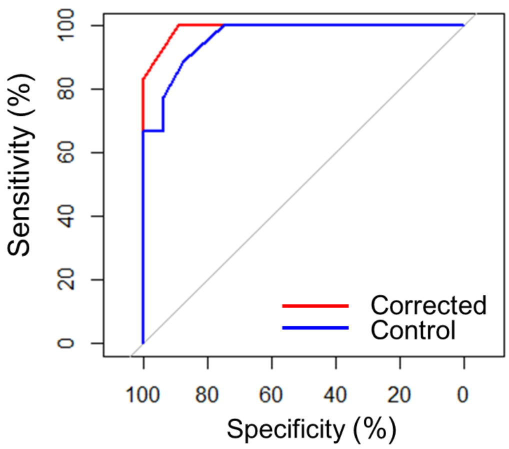

	Sn	Sp	PPV	NPV
Control	90	87	81	93
Corrected	100	93	90	100

Supplemental Figure 6. Schematic flow of the shuffling experiments using SW480 and NA12878 sequencing data. A library was generated separately for the DNA from each cell line. Afterward, the sequencing data was mixed according to three different ratios (SW480 1% shuffled, SW480 0.5% shuffled, and SW480 0.25% shuffled).

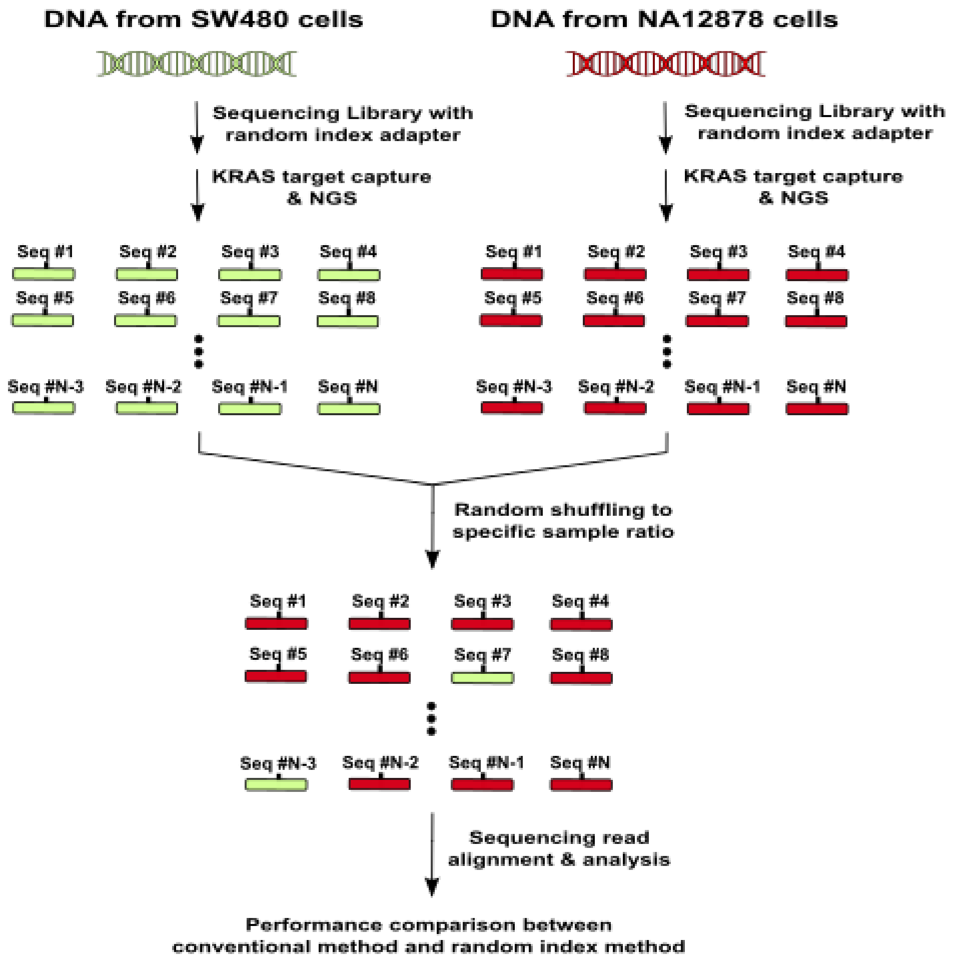

Supplemental Figure 7. Recovery of the depth in coverage in admixture samples (SW480 1% shuffled, SW480 0.5% shuffled, and SW480 0.25% shuffled) by applying the barcode and statistical error correction. Error bars represents mean +/- SD of duplicate experiments (n=2).

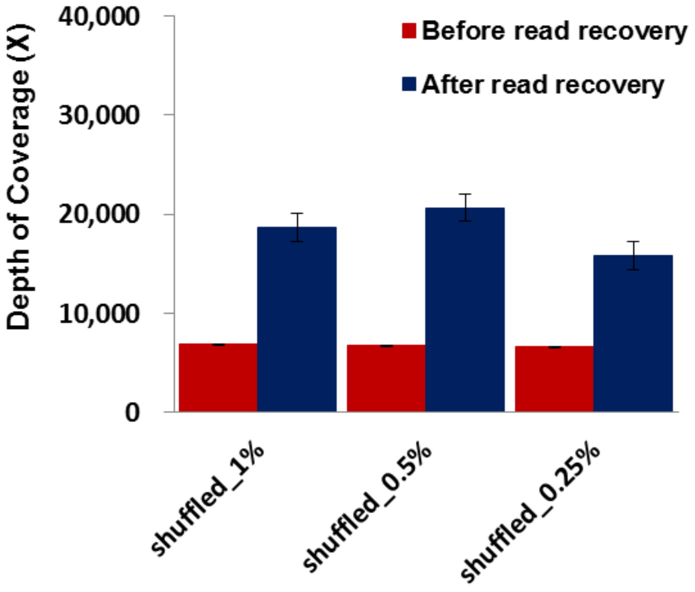

Supplemental Figure 8. Statistical error correction in three admixture samples (From top to bottom, SW480 1% shuffled, SW480 0.5% shuffled, and SW480 0.25% shuffled). The y-axis is the average allele frequency from two replicate experiments (n=2).

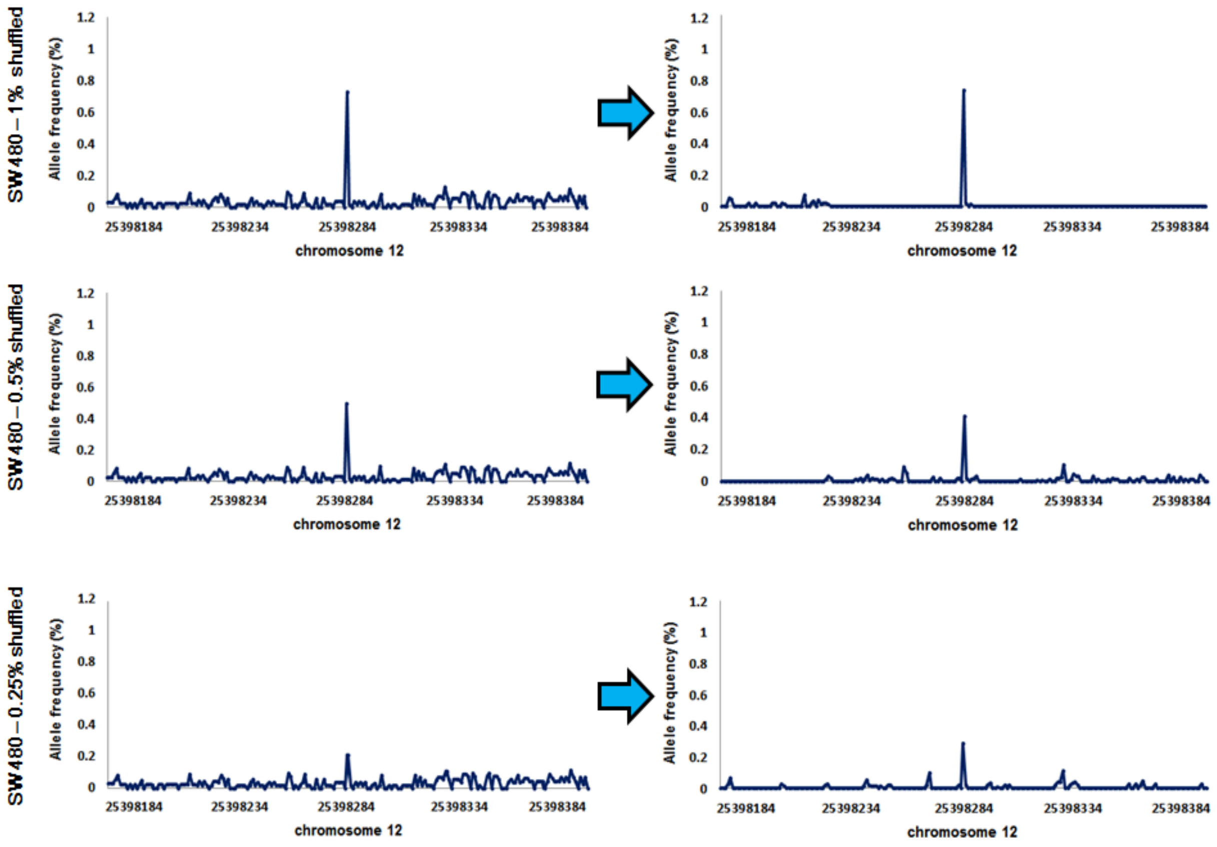

Supplemental Figure 9. Reduction of background-allele frequency by the application of a strategy to reduce background noise in five ctDNA samples. The allele frequency difference of the noise peaks was calculated after statistical error correction was applied.

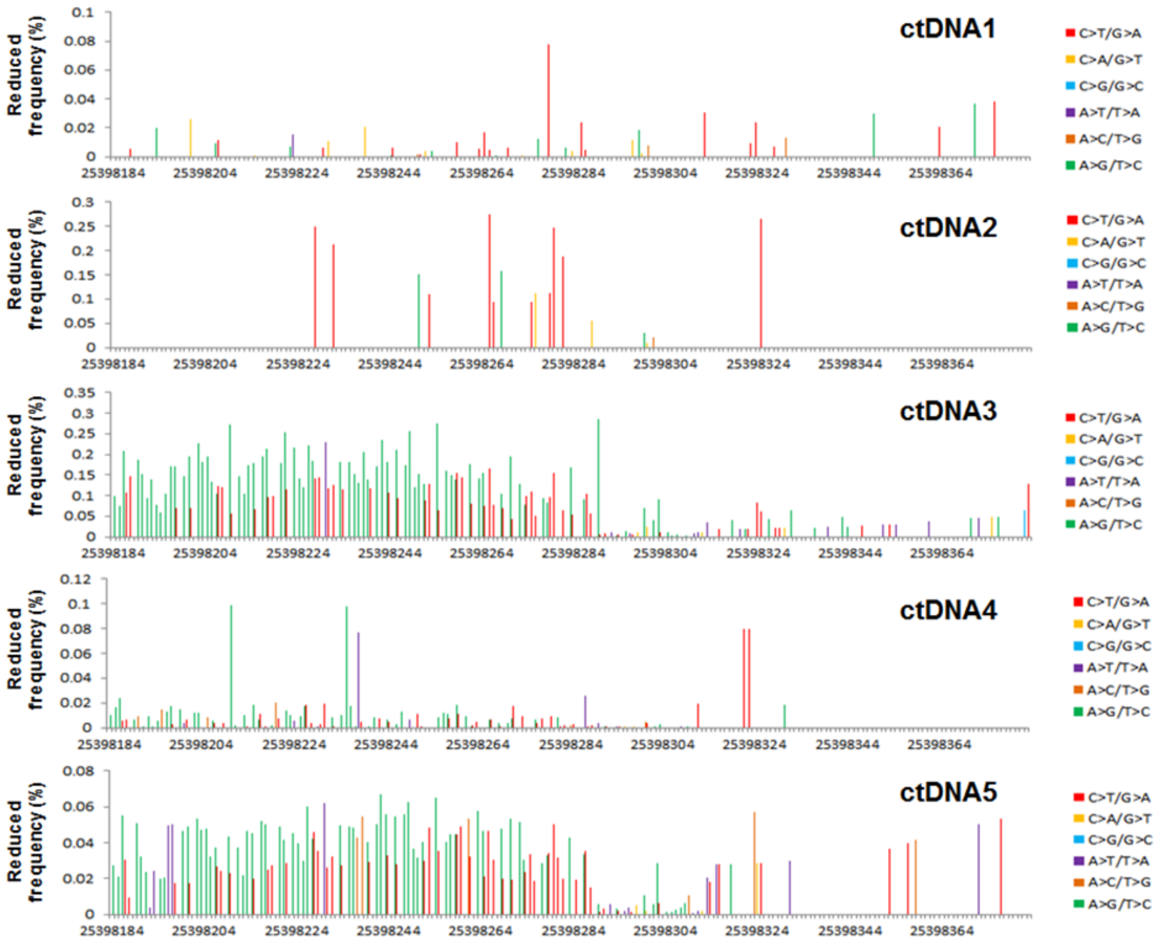

Supplemental Table 1. Clinical ctDNA sample sequencing statistics.

	Amount of ctDNA used	On-target	Total sequenced reads	Mean deduplicated	Mean depth recovered	Raw	Recovered rate (%)
ctDNA1	25ng	1.96%	20,322,978	1,424	5,173	11,920	62.7
ctDNA2	21ng	15.13%	4,564,958	2,721	4,373	10,876	63.1
ctDNA3	45ng	9.34%	6,218,270	2,695	7,380	11,585	63.7
ctDNA4	15ng	11.85%	34,995,838	2,405	3,109	11,919	62.8
ctDNA5	18ng	7.85%	38,798,268	2,872	3,730	11,919	62.8

Recovery rate was calculated as described in Supplementary Table 2 in iDES method (Newman et al, 2016). Briefly, recovered rate is calculated as hGE (Mean depth recovered) divided by the minimum of input hGE (330 times amount of ctDNA input) and the raw (mean non-deduplicated) depth.

Supplemental Table 2. Library preparation cost analysis.

Process	Name	Amounts	Cost	Used amounts per sample	Cost per sample	Total cost per sample	
Barcode adapter oligo synthesis	IDT oligo synthesis	200 µl	$90	2 µl	$0.90	$0.90	$300.13
Circulating tumor DNA extraction	QIAamp Circulating Nucleic Acid Kit	50 rxn	$1,500	1 rxn	$30.00	$214.40	
Sequencing library preparation	SPARK™ DNA Prep kit	8 rxn	$115	1 rxn	$14.40		
	Celemics™ KRAS gene target capture service	1 rxn	$170	1 rxn	$170.00		
Illumina sequencing	Illumina Hiseq 4000	1 lane	$3,500	1 lane (for 60 samples)	$58.33	$58.33	
Sample purification	AMPure XP beads	5,000 µl	$315	420 µl per sample library	$26.50	$26.50	
Total initial cost			$5,690				

Supplemental Table 3. Patient clinical information.

Sample ID	Gender	Age	Histology	Race	Stage	Smoking	Metastatic Organ	Largest Diameter of Metastatic Lesion (mm)
ctDNA1	M	66	Sigmoid colon	Adenocarcinoma	IV	Current, 23PY	Liver, Peritoneum	37
ctDNA2	M	65	Rectum	Adenocarcinoma	IV	Denied	Liver, Lung	100
ctDNA3	F	38	Sigmoid colon	Adenocarcinoma	IV	Denied	Liver, Lung	104
ctDNA4	F	55	Colorectal adenocarcinoma	Asian	IV	No	Presacral	39
ctDNA5	F	51	Colorectal adenocarcinoma	Asian	IV	No	Liver	27

Supplemental Table 4. Information on the *KRAS* target captured region.

Gene	Chromosome	Region start	Region end
KRAS	chr12	25,362,728	25,362,845
KRAS	chr12	25,368,370	25,368,494
KRAS	chr12	25,378,547	25,378,707
KRAS	chr12	25,380,167	25,380,346
KRAS	chr12	25,398,207	25,398,329
